# Large animal models of rare genetic disorders: sheep as phenotypically relevant models of human genetic disease

**DOI:** 10.1186/s13023-015-0327-5

**Published:** 2015-09-02

**Authors:** Ashish R. Pinnapureddy, Cherie Stayner, John McEwan, Olivia Baddeley, John Forman, Michael R. Eccles

**Affiliations:** Department of Pathology, Dunedin School of Medicine, University of Otago, P.O. Box 913, Dunedin, 9054 New Zealand; AgResearch, Invermay Agricultural Centre, Mosgiel, New Zealand; New Zealand Organisation for Rare Disorders, Wellington, New Zealand; Maurice Wilkins Centre for Molecular Biodiscovery, Level 2, 3A Symonds Street, Auckland, New Zealand

**Keywords:** Large animal model, genetic disease, sheep, human genome, ovine genome, DNA sequencing

## Abstract

Animals that accurately model human disease are invaluable in medical research, allowing a critical understanding of disease mechanisms, and the opportunity to evaluate the effect of therapeutic compounds in pre-clinical studies. Many types of animal models are used world-wide, with the most common being small laboratory animals, such as mice. However, rodents often do not faithfully replicate human disease, despite their predominant use in research. This discordancy is due in part to physiological differences, such as body size and longevity. In contrast, large animal models, including sheep, provide an alternative to mice for biomedical research due to their greater physiological parallels with humans. Completion of the full genome sequences of many species, and the advent of Next Generation Sequencing (NGS) technologies, means it is now feasible to screen large populations of domesticated animals for genetic variants that resemble human genetic diseases, and generate models that more accurately model rare human pathologies. In this review, we discuss the notion of using sheep as large animal models, and their advantages in modelling human genetic disease. We exemplify several existing naturally occurring ovine variants in genes that are orthologous to human disease genes, such as the *Cln6* sheep model for Batten disease. These, and other sheep models, have contributed significantly to our understanding of the relevant human disease process, in addition to providing opportunities to trial new therapies in animals with similar body and organ size to humans. Therefore sheep are a significant species with respect to the modelling of rare genetic human disease, which we summarize in this review.

## Introduction

Based on the number of genes involved, genetic diseases can be categorised into simple monogenic diseases, which tend to be rare, or polygenic diseases, which are much more common and multifactorial in nature. However, the principal difference between rare monogenic and common diseases is their mode of inheritance; only monogenic diseases follow a typical Mendelian pattern of inheritance [1]. The identification of novel mutations causing genetic disease has seen more progress in the last few years than in the previous twenty. This increased body of research has resulted in a wealth of information regarding rare diseases and is publicly available through projects like the HapMap project [2] (http://hapmap.ncbi.nlm.nih.gov/) and the Online Mendelian Inheritance in Man [3] (http://www.ncbi.nlm.nih.gov/omim). Identifying the underlying cause of disease is the first step in developing an effective treatment, and it can take several years to decades for an effective treatment of any given pathological disorder to be available. Until recently, identifying the causative gene in a given disorder could be a very time consuming process. Now with the advent of next generation sequencing (NGS) techniques, many large-scale genomic projects for the study of rare genetic diseases have come into effect. For instance, “The Undiagnosed Diseases Program” at National Institutes of Health (NIH), which started in May, 2008 [4], employed exome sequencing to screen patients who presented with previously undiagnosed genetic disorders and successfully diagnosed 39 patients in the first two years [4, 5].

### Rare genetic diseases

Several challenges exist in the study of rare genetic disease. Most of the monogenic diseases of unknown genetic cause are present at a very low rate in the human population. Among the nearly seven thousand rare genetic diseases that are known in humans, there are disorders so rare that their condition is limited to just a small number of reported cases in the world, for example Ablepharon-Macrostomia syndrome [6]. Researchers face significant difficulties in identifying patients with a particular rare disease of interest and obtaining sufficient numbers of patients for analysis. In addition, the total amount of funding available for research focused on rare genetic disease is scarce. Nevertheless, orphan diseases cannot be ignored, as collectively they pose a heavy burden on society. Seventy five percent of rare diseases affect children, and 30 % of these children die within the first five years of life. In addition, studying rare monogenic diseases significantly contributes to our understanding of both monogenic and polygenic diseases [7].

Following a conference involving 30 rare genetic disease groups in New Zealand, the New Zealand Organization for Rare Disorders (NZORD) was established as a charitable trust in the year 2000, and now has a network of 165 rare disease support and information groups. Its main aims are to help people affected by rare diseases, to create awareness about rare diseases, promote improvements to health care and social supports, and facilitate research into rare diseases by networking with researchers, affected patients, clinicians and policy makers. Information regarding various rare diseases is also made available publicly through their website, which links to other rare disease organizations across the world. NZORD promotes rare disease research through their charitable company the New Zealand Institute for Rare Disease Research Ltd (NZIRDR). NZIRDR has recently started a human tissue biobank, is maintaining a register of researchers in New Zealand with an interest in rare disease research, and is actively investigating prevalence and costs of rare diseases in New Zealand. It also has a database of animal models of human diseases, which occur naturally in New Zealand’s farm animal populations, and is planning a significant step forward in their genomics research initiatives through an active search for more large animal models. A complete list of all the animal models is available at the NZIRDR animal models web page [8] (www.nzirdr.org.nz/animal_models/our_models).

### Review: animal models and human disease

Many genes and their functions are highly conserved throughout the animal kingdom, and at the cellular level many biological processes are very similar between species, including cell proliferation, metabolism, and growth regulation. This homology across species is key to considering the possibility of studying diseases and their underlying molecular mechanisms in animal models of genetic disease. Indeed, animal models have been crucial in understanding both genetic diseases [9] and non-genetic diseases [10], alike. Furthermore, whole genome sequencing initiatives have now provided access to genome sequence information for approximately 60 species, including sheep and cattle, in addition to model organisms such as mice and zebrafish.

Animal models of human genetic disorders can provide excellent opportunities for pre-clinical studies to develop treatments for human disease. Choosing the appropriate animal model to address a biomedical research question is a complex process. Mice are very good models for biomedical studies, not least because of their short reproductive cycle, increasingly well-known genetics, ability to genetically manipulate embryos to create stable mouse lines, and small size [11]. Since the introduction of gene knockout technologies more than 25 years ago, thousands of gene knockout mouse models are now available. An ideal transgenic mouse model of human disease has a robust phenotype, well-defined abnormalities and pathological features that replicate the human disease well [11].

Examples of knockout mouse models of human disease include inherited neurodegenerative diseases, such as for example Huntington’s disease [12], Parkinson’s disease (PD) [13, 14], autosomal dominant polycystic kidney disease [15], and skeletal diseases such as Stickler syndrome [16]. While mouse models of human genetic disease have contributed significantly to the understanding of various genetic diseases, several factors limit mice as models of human disease. For instance, there are no reported cases of naturally occurring PD in any animal species to the best of our knowledge. Most PD mouse models are toxin induced, where chemicals like1-methyl-4phenyl-1,2,3,6-tetrahydropyridine (MPTP), paraquat or rotenone are used to produce nigrostriatial dopaminergic lesions. Although most of the toxin-induced mouse models affect mitochondrial function and/or create free oxygen radicals, none of them completely reproduce the pathology and clinical symptoms of the PD seen in humans [13]. In spite of their extensive use to study the disease, the results obtained using these models have not so far been successfully translated to the clinic. Furthermore, neurodegenerative diseases like PD and Alzheimer’s disease occur in an elderly population, usually over 60 years of age, making the short life span of rodents a limiting factor. The complexity and genetic heterogeneity of diseases such as PD have a huge impact on whether mouse genetic models successfully model the human disease. Mutations in the gene *Lrrk2* account for 5–6 % of patients with familial PD and 1–3 % of sporadic cases, but most *Lrrk2* transgenic mouse models showed minimal or no neurodegeneration [14].

There are many factors that impact on the therapeutic leap from genetic models to humans and this has been extensively reviewed for Huntington’s disease [17]. The shortcomings of mouse models for Huntington’s disease can be extrapolated to other genetic diseases with a late onset. Recently, a study investigated the genetic similarities of humans with inflammatory disease with different etiologies and concluded that although the conditions in humans share a high similarity, the corresponding murine models correlate poorly to the human conditions [18]. This study further indicated that there is a need for readdressing the current animal models and raised concerns over the feasibility of translational medical research using simple mouse models [18]. A possible solution for this is therefore to employ multiple animal models.

### Naturally occurring variants of human genetic diseases in animal counterparts

One of the most exciting land-marks in large animal research was marked by the successful cloning of the sheep named Dolly in 1996 [19]. This was a major leap forward in transgenic research and has led to the successful cloning of thousands of animals from 20 different species. There are many viable applications for this technology ranging from creating high-value farm animals, to producing transgenic animals or creating animal models for research.

Scientific awareness of the benefit of using large animal models in genetic research is increasing, as large animal models are more similar to humans in relation to size, lifespan, and certain clinical signs and physiology, than rodent models. They also have had a long history of use in medical research. Over the past few decades, large animal models, including sheep, have played important roles in preclinical trials [20], resulting in significant progress in the understanding of the pathological mechanisms of both complex and Mendelian genetic diseases. With the current lack of success in clinical trials, with less that 20 % proceeding past Phase II [21] the relevance of large animal models for pre-clinical work may become more apparent.

Progress in understanding genotype-phenotype relationships in human patients, has been facilitated by the study of animals with mutations in orthologous genes. Such animal orthologs are an important aid to the development of specific gene therapies for these disorders. There exist many animal models where naturally occurring disease-causing mutations that are homologous to mutations in the equivalent human disease have been discovered. Animal models that closely match the disease causing mutation in humans, like the *Cln6* ovine model of Batten disease (BD) [22], can be particularly useful in determining the pathophysiology of disease, and for devising new therapeutic strategies for diseases. Human genetic diseases such as Sandhoff disease (a lipid storage disorder) have been spontaneously found in dogs [23, 24] cats [25–28] and pigs [29–31]. Among large animal models, dog and pig have been important model species [32, 33]. However, livestock models such as sheep may offer some advantages for developing new large animal models of genetic disease.

### Sheep as a model of human genetic disease

Sheep are feasible large animal models for genetic studies for a number of reasons. Sheep are relatively outbred and much more human-like than mice, due to their similar size, and their closer genetic and physiological composition. In addition, because of this similarity sheep brains are similar in organization to non-human primates [34].

In New Zealand, sheep are ubiquitously farmed, domestic animals and therefore make cost-effective large animal models, with highly efficient agriculture systems already established. Their easy maintenance and acceptance of human handling saves both time and money. Sheep are also more socially acceptable animal models for research as compared to dogs. They are less expensive to purchase and manage than many closely related species such as cattle, particularly in New Zealand, which has a very large sheep population of approximately 30 million sheep. Using livestock such as sheep for medical research reflects the complexity of outbred populations [35].

In addition to cloning, large animal models for inherited diseases such as sheep can be established from naturally occurring variants. Sheep and cattle animal disease models frequently have mechanisms of disease similar to humans at both the molecular and morphological level [36]. There are strong founder effects in the various sheep breeds, well-established and recorded sheep studs, and well-documented phenotypic records. Regarding disease-causing mutations, the incidence of homozygous affected individuals is the square of the frequency of the disease allele. Therefore many deleterious recessive mutations may accumulate to a sizeable frequency, and yet homozygous affected animals may be infrequently seen in flocks, especially because maintenance of production values in stud flocks requires stringent culling, and affected or diseased lambs would generally not survive this. As a result, and because most sheep production systems in New Zealand utilize outbred animals, these homozygous animals may very seldom be seen on production farms. By tracing heterozygous carriers in stud flocks, it is relatively easy to find farms where a relatively high carrier frequency exists. These can be identified and a research flock established of carrier animals (for example, see polycystic kidney disease sheep below).

Sheep models for many inherited disorders already exist, such as those for inherited cataracts [37], achromatopsia [38], hemophilia [39, 40] and many others [8], and some have been successfully used in testing therapies for these diseases. A review in 2012, globally of sheep, cattle and goats, of traits for which causal gene variants were known [41] listed 14 autosomal recessive diseases analogous to diseases such as Batten disease, Ehlers-Danlos syndrome, inherited rickets and Gaucher disease. Most of these mutations were Single Nucleotide Polymorphisms (SNPs). For example, a spontaneous animal model for McArdle disease has been identified in sheep [42] caused by a SNP. There are several well-established sheep models of genetic disease that have made a significant impact on our understanding of particular diseases. In the following paragraphs we present several naturally existing sheep models of human disease.

### Sheep models of juvenile neuronal ceroid lipofuscinoses [Batten disease, JNCL, Juvenile NCL, Spielmeyer-Vogt disease] (ORPHA79264)

The neuronal ceroid lipofuscinoses (NCL) are a group of recessively inherited lethal neurodegenerative diseases. The disease in humans usually presents in previously normal children at around 5–10 years of age, with an incidence of 1 in 12500 live births [43]. Clinically, affected individuals are characterised by brain atrophy, vision loss due to retinopathy, seizures, and decline of mental and motor capacities, leading to premature death in the late teens or early adulthood. Accumulation of lysosome-derived storage bodies in neurons and in other cells types is a hallmark of this disease. Studies show that this disease is caused by mutations in at least 13 different genes, designated *CLN1-CLN8, CLN10-CLN13, CLCN6* and *SGSH* [44]. A large number of naturally occurring animal models that have mutations in the *CLN* genes have been described (www.ucl.ac.uk/ncl)^44^, including three sheep models. Mutations in CLN5 and CLN6 [9, 22, 34, 45–48] have been identified in New Zealand and Australian sheep, and affected animals share close clinical and pathological characteristics with the human NCLs. Research in these flocks over the last 40 years has led to many advances in the understanding of Batten’s disease, such as the identification of the “lipofuscin-like” storage material, determining the regional nature of the cortical atrophy and its close association with neuroinflammation, and more recently the ability to trial therapeutic approaches using drug and gene therapies [34].

### Jacob sheep, a Tay-Sachs disease model [GM2 gangliosidosis, B, B1 variant Hexosaminidase A deficiency] (ORPHA845)

GM2 gangliosidosis variant B, or Tay-Sachs disease (TSD), is marked by accumulation of G2 gangliosides due to hexosaminidase A deficiency. The incidence of the disease in non Ashkenazi Jewish populations is 1/360,000 and in the Ashkenazi Jewish population, the incidence of TSD is 1 in 3,600. TSD is a genetically heterogeneous rare autosomal recessive neurodegenerative disease with aggregation of Gm2 ganglioside in the nerve cells of the central nervous system [49]. Clinical symptoms include developmental delay, visual inattention, and seizures, which result in a vegetative state by 2 years of age. This disease results in death at around 3 to 5 years of age in children, due to a disproportionately enlarged brain caused by neuronal storage of the ganglioside [49].

In 2011, a group of researchers published a report showing the results of biochemical and molecular genetic analyses of four Jacob lambs diagnosed with TSD. The lambs, which were from the same farm in Texas, were investigated by the Texas A&M University veterinary hospital over a three-year period [50]. Detailed physical and histological examinations of the brains have given important insights into this genetic disease. The sheep were categorized into infantile, late infantile, juvenile and adult onset forms. The infantile sheep exhibited progressive clinical signs similar to the milder condition of TSD in humans. However, histopathological findings from the brain showed a high level of similarity to the human TSD patients. In contrast, attempts to create a TSD mouse model by gene knockouts have not been entirely successful [51,52]**.**

### A sheep model of Gaucher disease [acid beta-glucosiase deficiency, glucocerebrosidase deficiency] (ORPHA355)

Gaucher disease (GD) is a lysosomal storage disorder encompassing three main forms (types 1, 2 and 3), a fetal form and a variant with cardiac involvement (Gaucher disease - ophthalmoplegia - cardiovascular calcification or Gaucher-like disease). The overall prevalence is approximately 1/100,000. Lysosomes play a major role in living cells with functions such as constitutive recycling and autophagy [53]. Although over 300 different disease-causing mutations have been described in humans for Gaucher disease (GD), the lysosomal storage disorders in general are still relatively poorly understood.

GD is an autosomal recessive disorder, and is the most common lysosomal storage disorder with a wide range of clinical presentations. Mutations in the gene encoding β-glucocerebrosidase cause this disease. The three sub-types of GD are characterized by neurologically related problems with symptoms ranging from cytopenia, to anemia to bone disease in the patients. Naturally occurring GD has been identified in animals such as mice [54, 55] and pigs [56]. Although a sheep model of GD had been described previously in the 1960’s [57], a more recent naturally occurring variant of GD in a Southdown flock of sheep was identified and described in Australia [53]. Further structural analysis showed that the gene was highly conserved between sheep and humans and shared a high structural similarity. Further investigations including biochemical and histopathological examinations showed that the affected neonatal lambs displayed severe clinical signs that were highly similar to the most severe and very rare form of this disease, GD type-2 [53]. Two mutations were identified following a mutational screening and further analysis showed that one of the two missense mutations caused the severe phenotype of this disease [58].

### A sheep model of the recessive polycystic kidney disease, Meckel syndrome [Meckel-Gruber syndrome] (ORPHA564)

Meckel syndrome (MKS) is a rare, lethal, genetic disorder characterized by occipital encephalocele, large polycystic kidneys, and polydactyly as well as associated abnormalities that may include cleft lip/palate, cardiac and genital anomalies, central nervous system (CNS) malformations, liver fibrosis, and bone dysplasia. Incidence has been estimated as anywhere between 1 in 3000 to 1 in 140, 000, depending on the population studied [59].

Polycystic kidney diseases (PKD) are the most common inherited kidney disorders, in which many cysts form in the kidneys and other organs. The exact mechanism of how cyst formation in the kidney is triggered still remains to be understood [60]. In addition to the dominant form of PKD (ADPKD), there are many less common forms of PKD, including autosomal recessive polycystic kidney disease (ARPKD) with an incidence of approximately 1 in 20,000 to 1 in 40,000 live births, including MKS, which mainly affect children and infants.

**Fig. 1:**
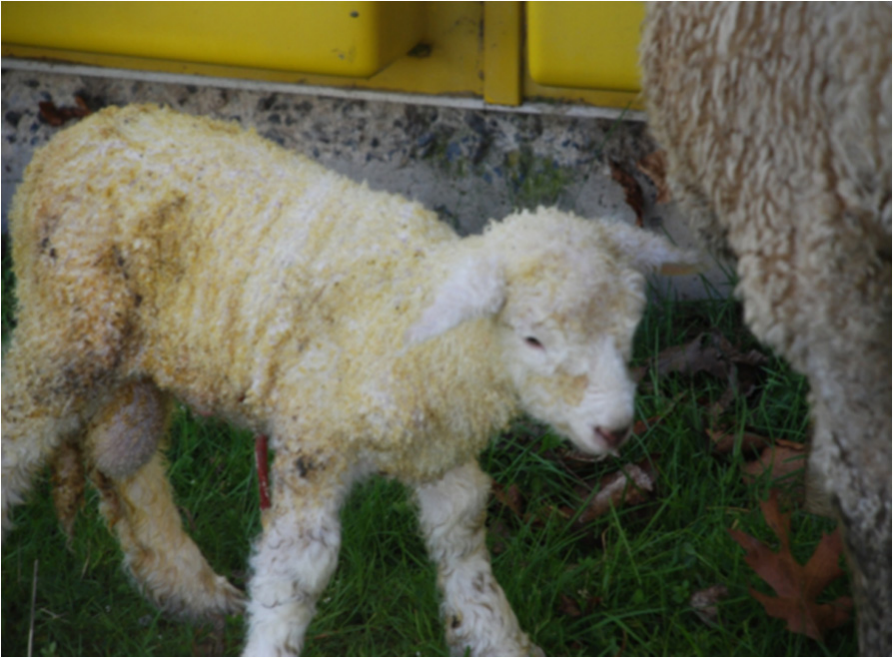
Photograph of an affected newborn lamb with a recessive polycystic kidney disease

Previously, we and colleagues have described a recessive form of PKD in two flocks of New Zealand sheep [61]. These two different breeds of sheep have been further characterized, and the recessive PKD in affected newborn lambs were found to have phenotypic characteristics similar to MKS, with a shared genetic abnormality in the 2 flocks (manuscript in preparation). Lambs with this disorder usually die perinatally, although we have observed that affected lambs are very occasionally born alive (Fig. 1). MKS is invariably fatal in the perinatal period [62].

### The sheep genome

One of the driving forces behind the success of small animal models like mice and zebrafish is the availability of their completely sequenced genome. Therefore, a major advantage in the genetic characterization of domestic farm animals is to have completely characterized genomes. An international consortium has recently completed a fully annotated sequence of the sheep genome [63] which has been made available to the public on the University of California Santa Cruz website (http://genome.ucsc.edu/cgi-bin/hgGateway). Furthermore, the availability of Ovine and bovine SNP chips has already impacted various commercial and academic research projects in Australia and New Zealand, and large amounts of data concerning single nucleotide variants in more than 55,000 sheep have already been generated. Using this SNP information together with current genomic technologies and methods, *in silico* identification of disease–causing mutations and embryonic lethal variants is possible. This, together with the vast amounts of phenotypic data that have already been collected by the NZ sheep industry, allows the possibility of systematic screening for the identification of putative disease-causing variants. Furthermore, the sheep industry in New Zealand has pedigree information on millions of animals, including those that have genotype information. Therefore there is infrastructure in the industry that can allow animals or flocks of animals harboring specific gene variants of interest to be easily identified and sourced in the population.

### NGS and the identification of new sheep models of disease

Since its invention in the 1970’s by Frederick Sanger, DNA sequencing has promulgated a major revolution in genetic research. The human genome project, which began in 1990, took 13 years and cost $2.7 billion to completely sequence the first human genome. The information gained through the completion of this project has played an important role in studying various monogenic and complex polygenic diseases. With the advent of NGS technology, it now takes approximately 3 days to completely sequence a human genome and costs only a few thousand dollars**.** This has provided the ability to carry out experiments that were previously unfeasible, and indeed NGS techniques have revolutionised the way scientists approach solving complex genetic problems in many fields of genomic research including microbiology, oncology, genetics and virology [64]. Moreover, equivalent efforts have also been carried out by the NIH (USA) and the U.S. Department of Agriculture, which has resulted in a completely characterized bovine genome sequence [65](http://bovinegenome.org/), and this therefore provides the opportunity for comparative genomic analysis between ovine, bovine and human.

The cost and time for NGS DNA sequencing has been lowered by orders of magnitude, and so it is now feasible to screen populations of sheep for genetic variants that resemble human genetic diseases. Indeed, this work is already well underway as part of production value surveys for variables associated with commercially important attributes, such as parasite resistance, growth rates and wool production, and with help from NZORD and collaborating organisations early-stage experiments have been started.

### Conclusions and future directions

The use of a diverse range of animal models, covering all aspects of disease manifestations at both the genotypic and phenotypic level allows for greater insights into disease mechanisms. The identification of natural variants of genetic disease in sheep is a first step towards the possibility of successfully establishing new sheep models for human genetic diseases. Indeed, ovine models of genetic disease such as the GD model have shown their worth as excellent pre-clinical models. Clearly, NGS will continue to play a role in the characterization of spontaneous genetic diseases arising in sheep, and will help to generate new ovine models of human genetic disease. However, it is now feasible to screen populations of sheep for genetic variants that resemble human genetic diseases, and with an appropriate investment in sequencing and bioinformatics resources, one possible future direction could be to use this NGS approach as a way to search for, and via carrier-carrier crosses, generate new ovine models of human genetic disease. The adoption of this approach could potentially result in a many-fold expansion of the numbers of ovine models of human disease, which would then help to realize both human and animal therapeutic benefits.

## Abbreviations

BD: Batten disease
GD: Gaucher disease
TSD: Tay-Sachs disease
NGS: Next generation sequencing
NIH: National Institutes of Health
NZORD: New Zealand Organization for Rare Disorders
NZIRDR: New Zealand Institute for Rare Disease Research
PD: Parkinson’s disease
MPTP: 1-methyl-4phenyl-1,2,3,6-tetrahydropyridine
NCL: neuronal ceroid lipofuscinosis
PKD: polycystic kidney disease
ARPKD: autosomal recessive polycystic kidney disease
ADPKD: autosomal dominant polycystic kidney disease
CNS: central nervous system
SNP: Single Nucleotide Polymorphism
